# Molecular Detection of *Aggregatibacter actinomycetemcometans* and *Porphyromonas gingivalis* in Children with Cleft Lip and Palate

**DOI:** 10.3390/dj14100635

**Published:** 2026-10-04

**Authors:** Shayma Abdullah Hanoon, Hiba Ahmed Saeed, Aseel Taha Khaudhair, Hind Thyab Hamid, Rusal Saad Ahmed, Hella Thamer Zaidan, Mohammed Nahidh

**Affiliations:** 1 College of Dentistry, Al-Muthanna University, Samawah 66001, Iraq; shayma.abdullah@mu.edu.iq (S.A.H.); hibaahmed@mu.edu.iq (H.A.S.); 2 College of Dentistry, Tikrit University, Tikrit 34001, Iraq; aseeltaha@tu.edu.iq (A.T.K.); hind.thamid@tu.edu.iq (H.T.H.); rusul.s.ahmed23@tu.edu.iq (R.S.A.); hella.t.zaidan23@tu.edu.iq (H.T.Z.); 3 College of Dentistry, University of Baghdad, Baghdad 10001, Iraq

**Keywords:** cleft lip and palate, periodontal pathogens, *Porphyromonas gingivalis*, polymerase chain reaction

## Abstract

**Background:** Children having cleft lip and palate (CLP) are predisposed to periodontal disease because of the anatomical and functional changes which predispose to plaque deposition and dysbiosis of microbes. Nonetheless, little is known about the molecular evidence of early colonization of crucial periodontal pathogens in this group of individuals. **Objective:** To identify *Aggregatibacter actinomycetemcomitans* and *Porphyromonas gingivalis* in children with CLP and to assess the relationship between them and their demographic, cleft-related, and periodontal clinical characteristics. **Methods:** The study was a cross-sectional study involving 110 children between the ages of 6–15 years of age with confirmed CLP. The subgingival plaque samples were obtained and analyzed by species-specific polymerase chain reaction (PCR). Clinical periodontal parameters were noted and the relationship between pathogen detection and clinical variables were evaluated using multivariate logistic regression analysis. **Results:** *A. actinomycetemcomitans* and *P. gingivalis* were found in 47.3% and 35.4% of the participants and both *Porphyromonas* and *Aggregatibacter* were co-colonized in 21.8%. There was a high age dependency and the highest prevalence of the pathogens was observed in children with both cleft lip and palate. The conditions of dual colonization were linked to much greater plaque index, gingival inflammation, and probing depth values (*p* < 0.001). Combined CLP phenotype, age 12 years and moderate-high accumulation of plaque were found to be independent predictors of detection of pathogens. **Conclusion:** Cleft lip and palate children exhibit early and recurrent colonization by major periodontal infections particularly in the presence of higher levels of the plaque and more extreme cleft morphology. Preventative intervention (in the form of early PCR-based screening of microbes) incorporated into multidisciplinary cleft treatment could offer specific preventive treatments and decrease long-term periodontal morbidity in this population at risk.

## 1. Introduction

Cleft lip and palate (CLP) are two of the most common congenital craniofacial defects in the world and they are due to the lack of complete fusion of the facial processes in the early stages of embryo development. Children with CLP have feeding problems, speech disorders, and abnormal development of faces, as well as significant problems with maintaining their oral health, which begin in early childhood and continue throughout their lives in most cases [1].

Cleft-related discontinuities in the anatomy have a tremendous impact on the oral ecosystem. Abnormal shape of alveoli, scar tissue due to repeated surgical operations, misaligned or absent teeth, oro-nasal fistulae, and long-term orthodontic care are some of the factors that greatly affect effective plaque control [2]. These factors facilitate the growth of biofilms, mechanical self-cleaning, and provide sheltered anaerobic ecological niches that prefer colonization by pathogenic microorganisms. Therefore, children who have CLP demonstrate greater plaque retention, gingivitis, and premature periodontal alterations in comparison to their non-cleft counterparts despite being given similar oral hygiene guidelines [3].

*Aggregatibacter actinomycetemcomitans* is a Gram-negative facultative anaerobe. It is usually associated with rapidly progressive periodontitis (Grade C), formerly aggressive periodontitis, which is strongly linked with aggressive and early-onset periodontitis, especially in children and adolescents [4,5]. *A. actinomycetemcomitans* has been demonstrated to be an early colonizer, which is a foregoing indicator of subsequent periodontal breakdown and can be seen as a key risk indicator.

Conversely, *Porphyromonas gingivalis* is a Gram-negative obligate anaerobe that may have a disproportionate impact on the composition and pathogenicity of the oral microbiome, and acts as a keystone pathogen [6]. Its existence has been continuously associated with generalized periodontitis and has been also involved in systemic inflammatory disorders, highlighting its extended clinical applicability.

This has been shown to persist through adolescence, as evidence shows that colonization by *A. actinomycetemcomitans* and *P. gingivalis* in childhood is not a short-term event; early patterns of microbial colonization may persist into adulthood and significantly increase the risk of periodontal loss of attachment and destruction of alveolar bone [7,8]. As a result, these pathogens in high-risk pediatric groups need to be identified in a timely manner.

These two specific pathogens were selected for many reasons. The first reason is that *A. actinomycetemcomitans* is the primary pathogen in periodontitis due to its secretion of potent virulence factor that destroys the immune cells and causes rapid bone loss in children, while *P. gingivalis* utilizes gingipains that degrade tissue structural protein and suppress immunity, driving local and systemic inflammation. Secondly, children with CLP have dental anomalies and scar tissue, which, in turn, create retentive areas for a low oxygen microenvironment, allowing the colonization of obligate anaerobes and facultative anaerobes. The last and most interesting reason is that the alveolar bone near the cleft is deficient, so the presence of these organisms will increase bone resorption and permanent damage that will prevent bone graft in the future, keeping in mind that they are not the only pathogens in the periodontal area.

The conventional methods of diagnosing periodontal pathogens have limitations, such as low sensitivity, long turnaround time, and anaerobic microorganisms being so fastidious that they can be difficult to work with when using culture-based methods and clinical indices. Such constraints are especially strong among pediatric patients, whose bacterial loads can be very insignificant, and whose disease symptoms can be less evident or absent. It has been overcome by molecular diagnostic methods, particularly polymerase chain reaction (PCR), which offers periodontal pathogen detection that is highly sensitive, specific, and fast and direct in clinical samples. Assays based on PCR make possible the detection of bacteria DNA at subclinical levels and hence the early determination of the risk before irreversible periodontal damage has taken place [9].

**Figure 1:**
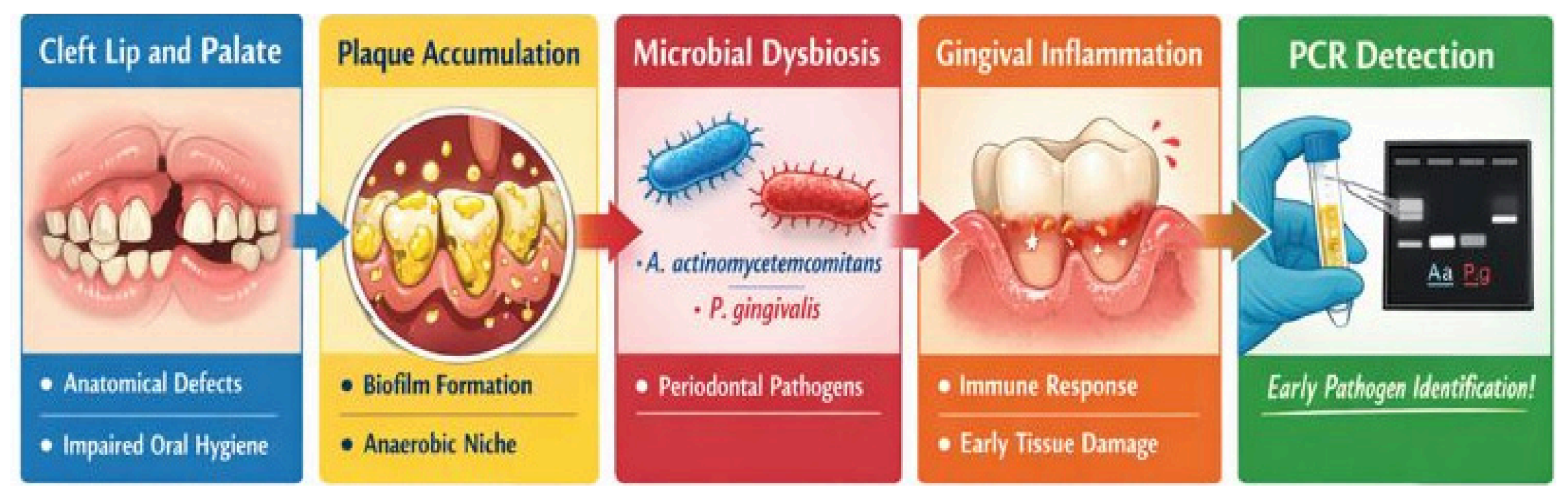
Conceptual framework illustrating cleft lip and palate-associated oral ecological disruption, early plaque accumulation, and PCR-based molecular detection of key periodontal pathogens.

A number of studies have shown the usefulness of PCR in identifying *A. actinomycetemcomitans* and *P. gingivalis* in children even in the absence of overt periodontal disease, and this favours the idea of microbial priming in periodontal pathology [10]. However, in spite of the already established predisposition of children with CLP to oral disease, there is limited molecular evidence on the prevalence and distribution of these significant periodontal pathogens in such a population. The vast majority of research available uses either clinical periodontal indices or heterogeneous microbiological surface techniques that could be underestimating the actual microbial load and postponing preventive action. This gap must be addressed in order to promote evidence-based oral healthcare measures that are specific to children with cleft lip and palate. Early detection of periodontal pathogens in the blood can inform both individualized preventive measures and the optimal timing of periodontal therapy, as well as decrease oral morbidity in this high-risk population in the long run. This type of PCR was selected because it targeted detection and qualitative presence of these organisms due to its technical simplicity, rapid workflow, and cost (Figure 1). It does not measure bacterial load and does not describe the complete microbial community, but it offered a resolution for our sample screening.

Hence, the objective of the current investigation is to molecularly identify *Aggregatibacter actinomycetemcomitans* and *Porphyromonas gingivalis* among children with cleft lip and palate by employing the use of PCR methods. The study will provide a better insight into the importance of microbial dysbiosis related to CLP, as well as contribute to the formulation of more specific preventive, diagnostic, and treatment approaches to support the future of long-lasting periodontal health outcomes.

## 2. Materials and Methods

### 2.1. Study Design and Setting

**Figure 2:**
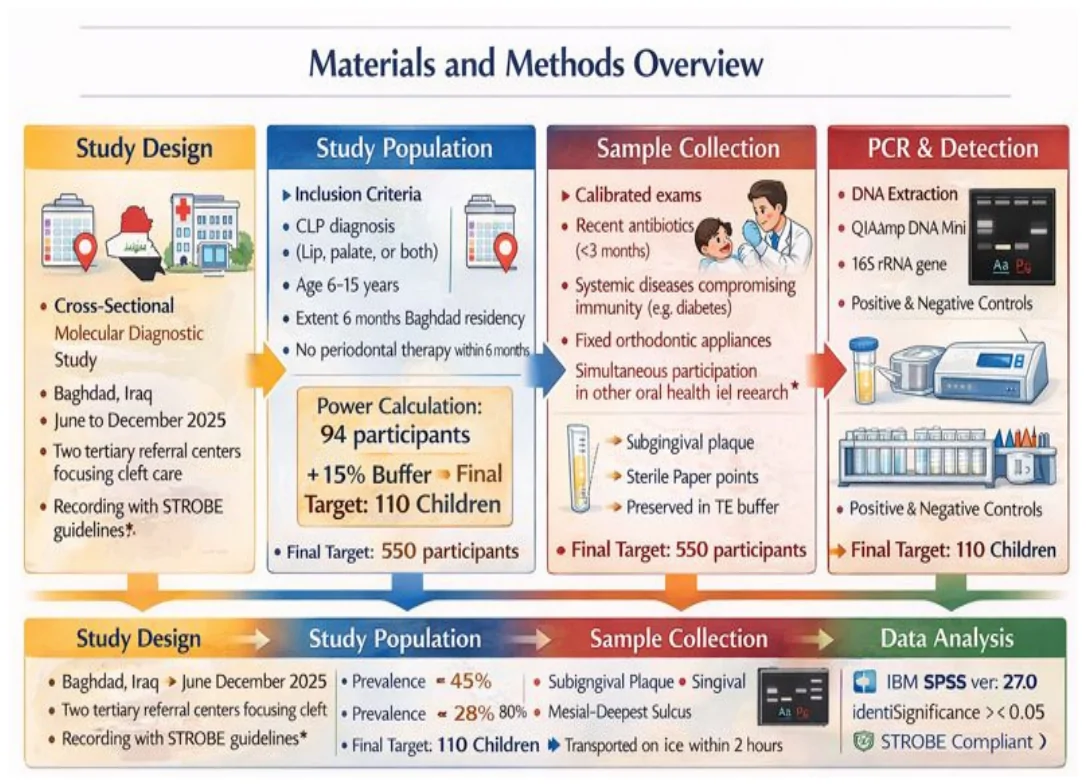
Study design and molecular workflow for periodontal pathogen detection in children with cleft lip and palate.

The study was a cross-sectional molecular diagnostic investigation and was carried out at Baghdad, Iraq, during the six-month period between June 2025 and December 2025, and was based on the Strengthening the Reporting of Observational Studies in Epidemiology (STROBE) guidelines. The intervention was carried out in two tertiary referral units in the field of pediatric dentistry and cleft lip and palate care that serve as key regional centers of surgical reconstruction, orthodontic therapy, and long-term multidisciplinary follow-up services of children in the affected group (Figure 2).

### 2.2. Study Population and Sample Size Determination

The sample of the study was a group of children between 6–15 years of age who had clinically and radiographically diagnosed cleft lip, cleft palate, or unilateral or bilateral cleft lip and palate. The subjects were sampled sequentially when they paid regular follow-up visits to reduce selection bias and to capture the representation of usual clinical groups. The sample size was estimated using the already reported rates of prevalence of periodontal pathogens in children, by assuming that the prevalence of *Aggregatibacter actinomycetemcomitans* and *Porphyromonas gingivalis* would be 45 and 28, respectively. The smallest sample size was computed to be 94 subjects using 95% confidence level and 80% statistical power. To reduce the effects of possible DNA extraction failure, sample degradation or participant withdrawal (which was expected at about 15%), the overall enrollment number of the sample was amended to 110 children, thus providing strong statistical power (Figure 2).

### 2.3. Eligibility Criteria

Inclusion Criteria

1. Confirmed diagnosis of cleft lip and/or palate, regardless of laterality or extent.
2. Age between 6 and 15 years at enrollment.
3. Continuous residence in Baghdad for at least 12 months prior to participation.
4. Absence of periodontal treatment, scaling, or root planing within the preceding six months.
5. Ability to cooperate with clinical examination and subgingival sampling procedures.

Exclusion Criteria

1. Systemic antibiotics use in three months before sample collection.
2. Existence of systemic, autoimmune, or immunocompromising diseases (e.g., diabetes mellitus, primary immunodeficiency).
3. Continued fixed orthodontic appliance treatment that is likely to confound the measurement of plaque or the accuracy of sampling.
4. Failure to give sufficient samples of the plaque or cooperate in examination.
5. Other oral health or periodontal research studies are underway.

### 2.4. Clinical Periodontal Assessment

The clinical check-ups were done by five qualified pediatric dentists, who had over eight years of clinical practice experience before the main study with 20 children not involved in the study group, under standardized conditions using portable LED lights and sterile intra-oral tools. Examiner calibration was done before the commencement of the study and reviewed at four-week intervals during the data collection period. Intra-examiner reliability showed a high level of agreement with a kappa coefficient of 0.87, which is above the established acceptability range. The status of oral hygiene was measured with the Simplified Plaque Index [11], and gingival inflammation was measured with the Modified Gingival Index [12], with bleeding response being noted at six sites per tooth. A UNC-15 periodontal probe was used to measure periodontal probing depth and clinical attachment level at six sites per tooth with measurements to the nearest millimeter. All data were recorded on standardized, pre-coded data collection instruments.

### 2.5. Subgingival Plaque Sampling Protocol

Subgingival plaque samples were taken at the deepest periodontal sulcus or pocket detected at clinical examination to achieve maximum sensitivity of pathogen detection. In cases where several sites had similar probing depths, the mesial surface of the tooth having the highest change was used as the sampling standard [13]. The sites of sampling were sterilized using sterile cotton rolls and dried by blowing on them over a five-second period to avoid excessive salivary contamination and to prevent causing tissue trauma. Subgingivally, sterile paper points (ISO size of 25–30) were inserted at the base of the sulcus and left in place for 20 s to enable sufficient biofilm uptake. A 500 µL TE buffer was placed in each paper point and transferred into a 1.5 mL sterile and nuclease-free microcentrifuge tube. The samples were kept on ice during a 2-h period after collection and kept at −20 °C, and storage should not exceed four weeks before DNA extraction (Figure 2).

### 2.6. Genomic DNA Extraction and Quantification

The bacterial genomic DNA was isolated with the use of QIAamp DNA Mini Kit (Qiagen, Hilden, Germany) according to the manufacturer’s protocol, and specific changes were made to suit the matrix of oral biofilm. These were the duration of enzymatic lysis (60 min at 56 °C), and mechanical lysis before and after using 0.1 mm zirconia bead-beating (30-s cycles, 3 cycles) to increase recovery of Gram-negative periodontal pathogens. NanoDrop 2000 spectrophotometer (Thermo Fisher Scientific, Waltham, MA, USA) was used to measure the DNA yield and purity. Acceptable samples were those with a DNA concentration of at least 50 ng/dL and a ratio of 260/280 absorbance between 1.8 and 2.0. To maintain the integrity of amplification, purified DNA was stored at −20 °C and analyzed within a period of eight weeks.

### 2.7. Polymerase Chain Reaction (PCR) Amplification

Molecular detection of *Aggregatibacter actinomycetemcomitans* and *Porphyromonas gingivalis* was done by the use of species-specific molecular methods, namely PCR, which is effective in cost and as a specific qualitative detector, but limited in quantitative measures compared with other types such as qPCR or shotgun metagenomic sequencing. This type used in our study is designed to detect the target species using conserved regions of the 16S rRNA gene. Prior known primers that have been validated in peer-reviewed literature were first synthesized commercially and are presented in Table 1.

PCR reactions were performed in 25 µL of final volume, containing template DNA (50–200 ng), 0.2–0.5µM of each species-specific primers, 1× PCR master mix from 2× concentrated stock, and nuclease-free water. An Applied Biosystems Veriti Thermal Cycler (Thermo Fisher Scientific, Waltham, MA, USA) was used under optimal conditions for amplification, first denaturation at 95 °C for 5 min, 35 cycles of denaturation (95 °C, 30 s), annealing (58 °C, 30 s), and extension (72 °C, 45 s), and finally extension (72 °C, 5 min). Both amplification runs used positive controls (*A. actinomycetemcomitans* ATCC 29523; *P. gingivalis* ATCC 33277), extraction blanks, and negative controls using only reagents to ensure assay specificity and remove contamination. After that, amplicons were separated by 1.5% agarose gel electrophoresis, stained, and visualized under UV transillumination.

### 2.8. Agarose Gel Electrophoresis and Visualization

PCR products were resolved by electrophoresis on 1.5% agarose gels prepared in 0.5× TBE buffer and stained with ethidium bromide or GelRed. Electrophoresis was conducted at a constant voltage of 100 V for 45 min, followed by visualization under ultraviolet transillumination using a ChemiDoc MP imaging system. Amplicon sizes were verified against a 100 bp DNA ladder, ensuring precise confirmation of species-specific amplification.

### 2.9. Quality Assurance and Reproducibility

Any molecular procedure was done in duplicate to each sample. Discordant samples were re-analyzed and concordant results were needed to classify the samples as final. There were also strict contamination control measures, such as physical separation of pre- and post-PCR work areas, unidirectional workflow, and regular environmental controls processing. There was no contamination that was identified as a result of the study period.

### 2.10. Statistical Analysis

Statistical programs were done with IBM SPSS Statistics version 27.0. The pathogen prevalence and the related 95% confidence intervals were computed using descriptive statistics. The chi-square test or Fisher exact test was used to test the associations between bacterial detection and demographic or clinical variables. Independent sample t tests or Mann tests based on the normality of distribution were used to analyze continuous variables. Multivariate logistic regression models were developed to determine independent predictors of the presence of pathogens, which controlled for age, sex, and plaque accumulation. All the statistical tests were two-tailed, and a *p*-value below 0.05 was taken as statistically significant. In situations where multiple comparisons were done, Bonferonni correction was used.

### 2.11. Equipment and Reagents Specification

All reagents, instruments, and software used in this study were clearly specified to ensure methodological reproducibility. Genomic DNA extraction was performed using the QIAamp DNA Mini Kit (Qiagen, Hilden, Germany) according to the manufacturer’s protocol. PCR amplification was carried out using a Veriti Thermal Cycler (Applied Biosystems, Foster City, CA, USA). DNA concentration and purity were measured using a NanoDrop 2000 spectrophotometer (Thermo Scientific, Wilmington, DE, USA).

Agarose gel electrophoresis was performed using a horizontal gel electrophoresis system (Bio-Rad, Hercules, CA, USA), and visualization of PCR products was conducted using a ChemiDoc MP Imaging System (Bio-Rad, USA).

All statistical analyses were performed using IBM SPSS Statistics version 27.0 (IBM Corp., Armonk, NY, USA).

## 3. Results

### 3.1. Participant Characteristics and Periodontal Status

One hundred and ten children who had cleft lip and/or palate were used in the final analysis. The quality and quantity of all the collected samples of subgingival plaque were adequate to proceed to PCR amplification, meaning that 100% of the samples were included in the analysis. The average age of the subject participants was 10.8 + 2.6 years (range: 6–15 years). The cohort was male (56.4% n = 62) and female (43.6% n = 48).

**Figure 3:**
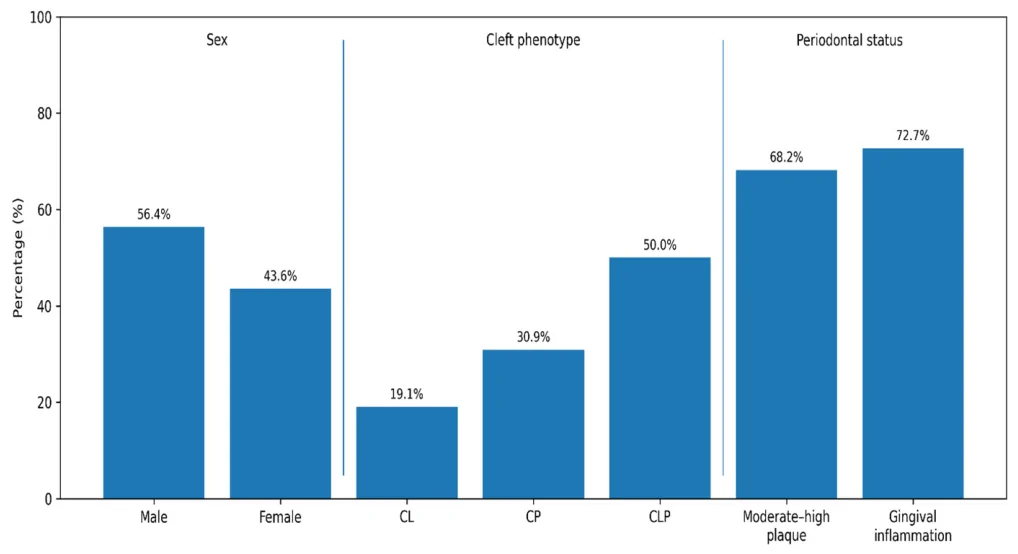
Demographic variables, cleft phenomenon distribution, and periodontal condition of the research sample (n = 110).

In regard to cleft morphology, combined cleft lip and palate (CLP) was the most common phenotype (50.0%), with isolated cleft palate (30.9%) and isolated cleft lip (19.1%) in second and third places, respectively. The clinical periodontal evaluation revealed a high amount of pathology: 68.2% of children showed moderate-to-high levels of plaque and 72.7% of children had clinical indicators of gingival inflammation (Table 2, Figure 3).

### 3.2. Molecular Detection and Co-Colonization Patterns

**Figure 4:**
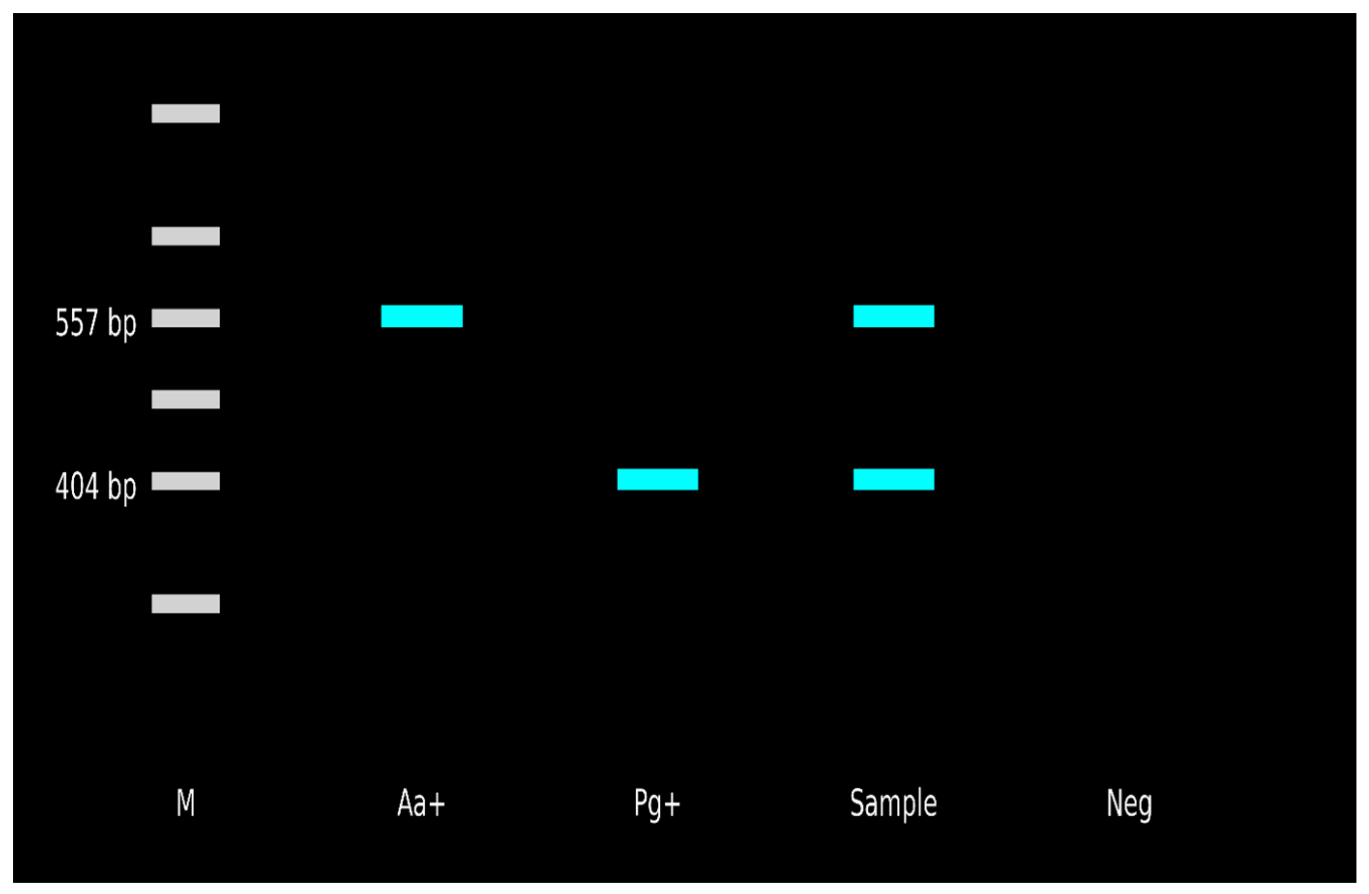
Representative agarose gel electrophoresis of PCR amplification products. Bands at 557 bp (*Aggregatibacter actinomycetemcomitans*) and 404 bp (*Porphyromonas gingivalis*) as well as a molecular weight marker (M), positive controls, one representative clinical sample, and the negative control are depicted. The Bright cyan band represents the DNA bands (PCR amplicons), while the Gray/white bands represents the DNA ladder/marker bands.

Molecular analysis using PCR (Figure 4) showed effective efficacy in the detection of both target periodontal pathogens. The presence of *Aggregatibacter actinomycetemcomitans* was found in 47.3% (n = 28) of the sampled individuals, whereas *Porphyromonas gingivalis* was present in 35.4% (n = 14) of the sampled individuals. Only 21.8 (n = 24) children were co-colonized with both pathogens (Table 3).

The percentage of *A. actinomycetemcomitans* was notably greater than that of *P. gingivalis* (*p* = 0.021), which may indicate the prior settlement of facultative anaerobic pathogens into the subgingival microbiota of pediatric CLP patients.

### 3.3. Distribution of Periodontal Pathogens by Age, Sex, and Cleft Phenotype

**Figure 5:**
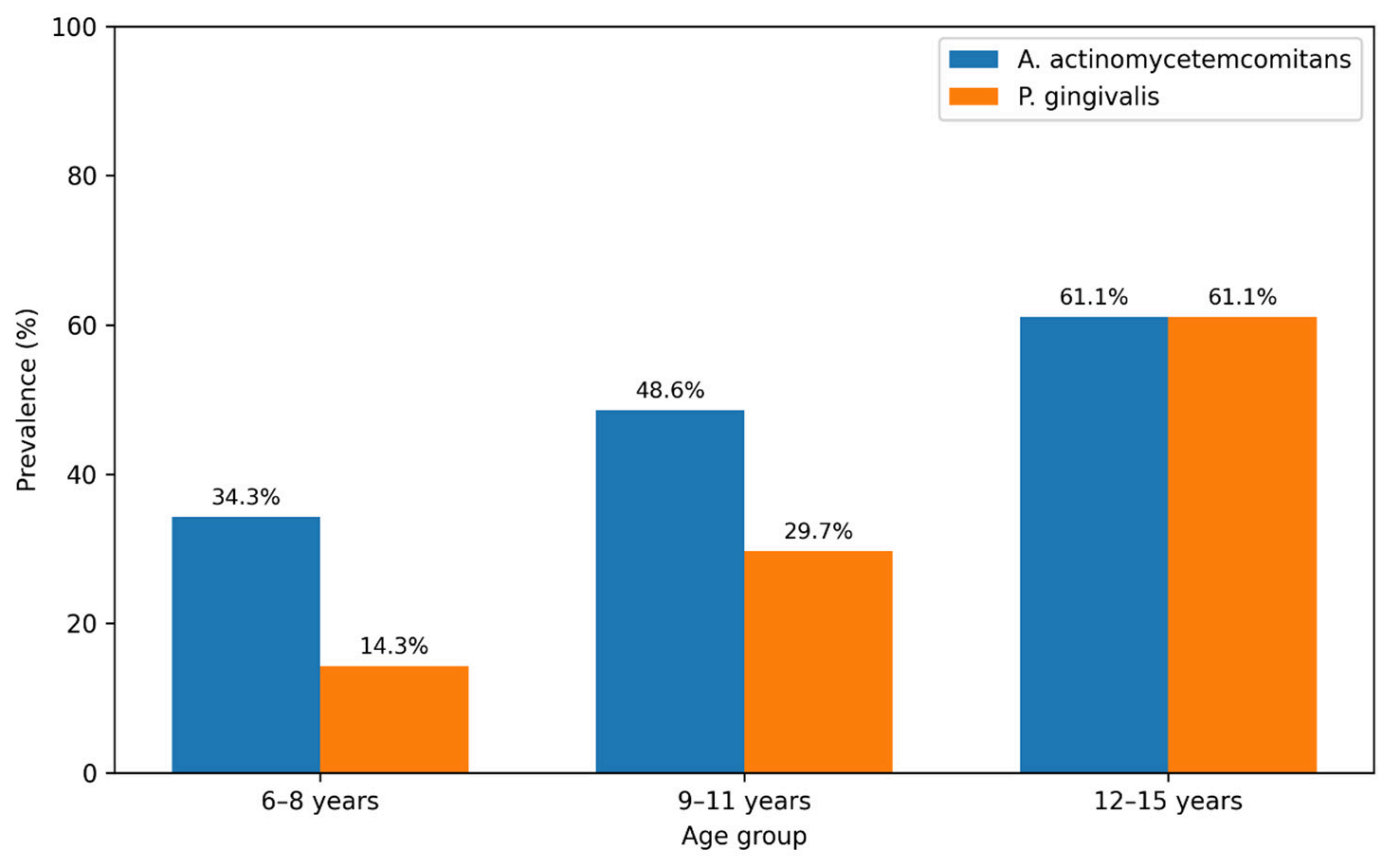
Periodontal pathogen stratification according to age.

The increase in pathogen detection was evident with age. Although *A. actinomycetemcomitans* was commonly observed in all age groups, *P. gingivalis* was observed to be more prevalent with aging, with peak prevalence rates in children aged 12–15 years (*p* = 0.004). Combined CLP phenotype was characterized by a statistically significant higher prevalence of both pathogens in children with combined types of clefts than isolated ones (*p* = 0.018). They did not find any statistically significant sex-based differences in either pathogen (Table 4, Figure 5).

### 3.4. Association Between Pathogen Presence and Clinical Periodontal Parameters

**Figure 6:**
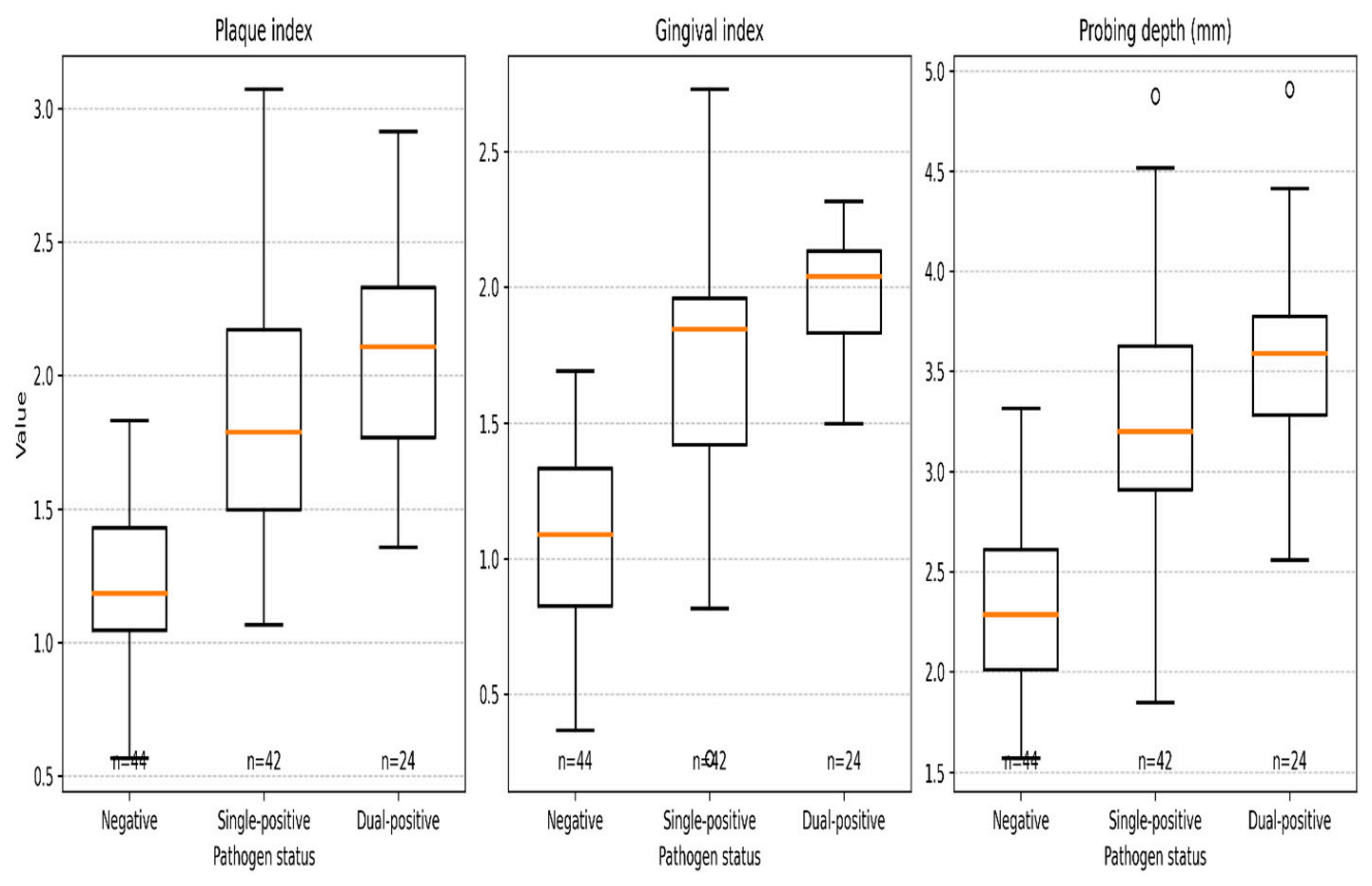
Clinical periodontal indices based on pathogen status. Box-and-whisker plot showing progressive deteriorations in the plaque index, gingivitis, and probing depth of the pathogen negative group, single-positive group, and dual-positive group.

Children that were positive for either or both periodontal pathogens had considerably poor clinical periodontal parameters. The values of mean plaque index, gingival index, and probing depth were increasingly dominant in single-positive and dual-positive groups relative to children who were not colonized with the pathogens (*p* < 0.001). Interestingly, children with dual colonization presented the greatest burden of inflammatory parameters, suggesting a possible synergistic effect of pathogens (Table 5, Figure 6).

### 3.5. Multivariable Predictors of Periodontal Pathogen Detection

**Figure 7:**
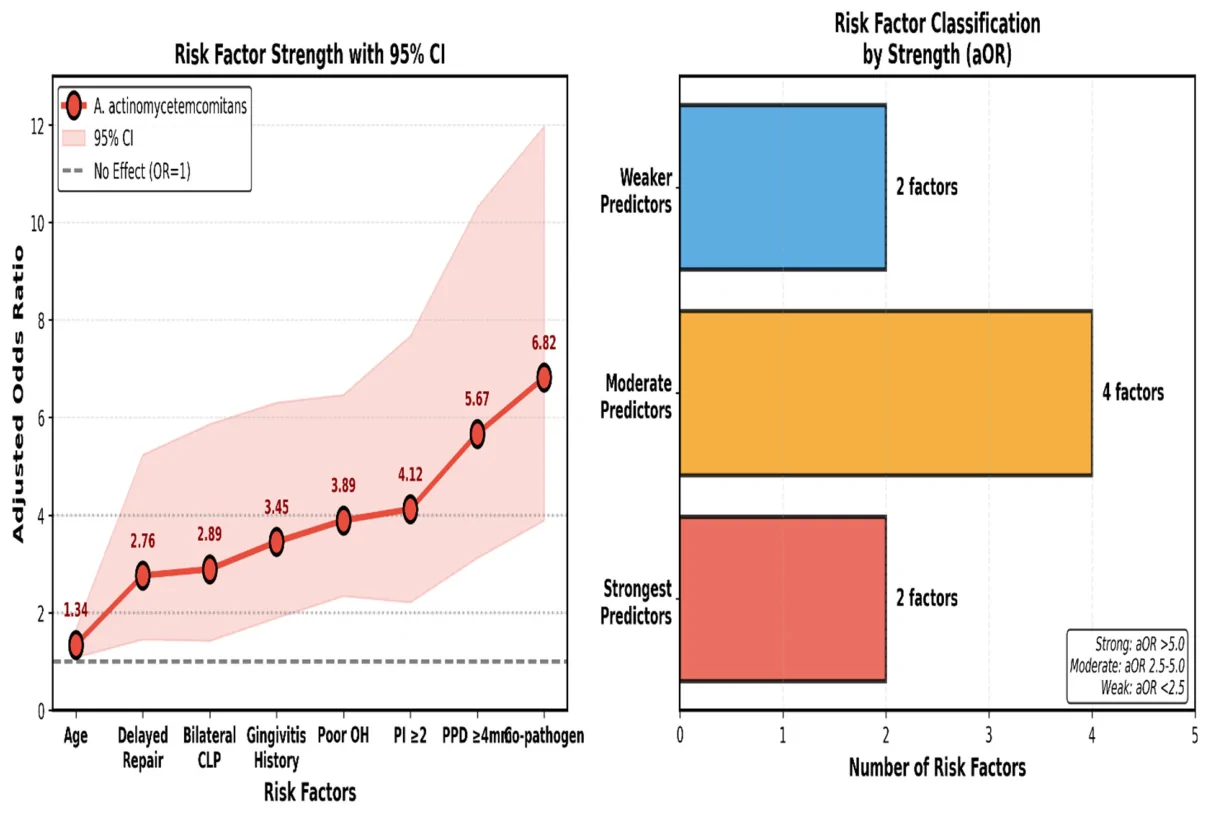
Adjusted odds ratios and strength-based classification of periodontal risk factors.

Multivariable logistic regression analysis found age 12 years and above, moderate-to-high plaque accumulation, and combined CLP phenotype to be independent predictors of periodontal pathogen detection. The odds ratio of 2.5 for carrying *P. gingivalis* in children aged ≥12 years increased 3.4-fold with high plaque levels, and dual colonization was also 4.6-fold higher (Table 6, Figure 7).

## 4. Discussion

The findings of the study give compelling evidence, both molecular and clinical, that infants who have cleft lip and palate (CLP) are characterized by early, frequent, and clinically relevant colonization by important periodontal infections. In particular, *Aggregatibacter actinomycetemcomitans* and *Porphyromonas gingivalis* are the pathogens that manifest themselves in this manner. The higher vulnerability to periodontal disease that has been frequently documented in CLP populations may be explained physiologically by the combination of PCR-based detection with thorough periodontal evaluation. The results of this study make it abundantly evident that the periodontal risk in children with cleft lip and palate is established at an early age, increases with age, and is highly impacted by the buildup of plaque and the severity of the cleft phenotypic.

The ecological niche of children with cleft lip and palate (CLP) is a unique environment of the oral cavity marked by anatomical discontinuities, scarring in places of surgery, and reduced oral activity, which offer difficulties in the effective management of biofilm. Longer period of plaque retention, low quality salivary clearance, and reduced self-cleansing ability are variables that contribute to the onset of early microbial dysbiosis [14,15,16]. Nevertheless, these methods are not comprehensive enough to describe the microbial pathways that underlie the cause of illness [14,15]. Past studies, which primarily targeted clinical outcomes, suggested that CLP patients experienced a higher rate of gingivitis and plaque. Despite the lack of clinical signs of widespread periodontal loss, the existing molecular evidence directly suggests the current presence of periodontal infections in a high proportion of children with cleft lip and palate.

The prevalence of *A. actinomycetemcomitans* that was observed in this group is consistent with its long-established role as an early colonist associated with periodontal disease in children and adolescents [17,18]. Its facultative anaerobic metabolism allows it to live in gingival sulci that are relatively shallow, and in juvenile biofilms, allowing it to become established within the oral cavity at an earlier stage. Moreover, its virulence factors, the largest of which are leukotoxin and cytolethal distending toxin, directly interfere with the activity of neutrophils and lymphocytes, thereby predisposing to the development of long-term inflammation and reducing the efficiency of the host immune surveillance system [5,8,19,20]. The importance of *A. actinomycetemcomitans* as a microbiological risk indicator has been emphasized by the reality that longitudinal investigations have demonstrated that early colonization by *A. actinomycetemcomitans* is a forerunner of future periodontal breakdown and clinical loss of attachment, and predictive of long-term periodontal breakdown [21,22].

*P. gingivalis*, for its part, exhibited a significant increase in prevalence dependent on age, and it was found to be more prevalent among older children. This pattern also complies with the generally accepted microbial succession model of periodontal disease, according to which obligate anaerobes emerge during the expansion of the incidence of gingival inflammation and the increasing anaerobic condition within the sub-gingival environment [23,24]. This trend is in line with this idea. It is clinically significant that *P. gingivalis* has been found during late infancy and early adolescence because of its possible ability to produce dysbiosis and immunological dysregulation even at low levels of abundance [25,26]. There is therefore a possibility that its premature presence can accelerate the development of gingivitis to severe periodontal disease among vulnerable, high-risk pediatric populations with CLP.

One of the most significant findings that came out of this study is the close relationship between dual colonization and deterioration of periodontal clinical parameters. Children with *A. actinomycetemcomitans* and *P. gingivalis* exhibited higher levels of plaque index and gingiva inflammation and probing depth of the periodontium than single-positive and pathogen-negative children. This observation lends credence to the idea that synergistic interaction of microbes, and not individual pathogens, causes periodontal disease [27,28]. It has also been shown experimentally that immune suppression caused by *A. actinomycetemcomitans* can enhance *P. gingivalis* colonization and that proteolytic enzymes of *P. gingivalis* can further undermine host defense activities and connective tissue architecture, increasing periodontal tissue destruction [29,30]. The fact that this kind of synergistic relationship is present at a pediatric age reflects the aggressiveness of periodontal risk among CLP populations.

The severity of cleft phenotype was evaluated as a significant predictor of microbial burden with children experiencing combined cleft lip and palate having very high prevalence of pathogens compared to those with isolated clefts. Greater cleft defects are linked with a higher level of anatomy, surgery, and a longer period of orthodontics, which leads to greater plaque retention and microorganism survival [31,32]. Though previous research proposed that there is a correlation between cleft severity and periodontal outcomes, discrepancies in the use of microbiological evaluation made conclusive findings impossible [33]. The current molecular evidence is more compelling evidence that combined CLP phenotype has an independent positive effect on the chances of colonization by periodontal pathogens.

The accumulation of plaque became the most potent independent predictor of pathogen identification and dual colonization than any of the factors that were examined. The significance of the mass and maturation of biofilms in the pathogenesis of periodontal disease is further reinforced with this finding [34,35]. The difference between a risk factor and non-modifiable determinants such as age or cleft phenotype is that plaque is much different and can be changed. It has been indicated that more preventative treatment that incorporates caregiver-administered oral hygiene and professional plaque may reduce periodontal inflammation in populations with CLPs to a drastic extent. The outcome of the present study, which relied on the use of molecular analysis, offers substantial evidence in favor of the implementation of such targeted preventive treatment at an early age. In the future, if the molecular findings are translated into daily practice in pediatric dentistry with the help of PCR based diagnostics, very sensitive detection of periodontal infections could be performed even in children who had few clinical signs. This enabled the removal of limitations that were linked to the traditional culture-based or purely clinical diagnostic methods. The availability of microbial risk could be detected before irreversible damage to periodontal tissue took place through the aid of molecular detection, which agrees with the principle of modern preventive and precision dentistry. This approach is particularly useful when dealing with pediatric groups, where early interventions offer the most significant scope for preservation of periodontal health in the long run. These findings should be interpreted with caution, as the cross-sectional design precludes causal inference and restricts the interpretation of results to associations rather than cause–effect relationships.

### Study Limitations

This study has several limitations that should be acknowledged. First, the absence of a non-cleft control group limits the ability to make comparative conclusions regarding increased susceptibility. Second, sampling was restricted to a single deepest periodontal site, which may not fully represent the overall oral microbiome. Third, PCR-based detection identifies the presence of bacterial DNA but does not provide information on bacterial load or metabolic activity. The study was limited also in that it didn’t examine and correlate the bacteria with different types of cleft lips and palate. In addition, the cross-sectional design precludes the establishment of causal relationships. Therefore, the findings should be interpreted as associations rather than indicators of active infection or disease progression.

## 5. Conclusions

This study presents evident molecular data that children with cleft lip and palate are already at high periodontal risk at a young age, as demonstrated by high concentrations of *Aggregatibacter actinomycetemcomitans* and *Porphyromonas gingivalis*. The age-associated increase in the detection of pathogens was highest in children with combined cleft lip and palate and moderate-to-heavy accumulation of plaque. The most common dual colonization was linked to the worst periodontal parameters, suggesting synergistic pathogenicity and speedy development toward periodontal dysbiosis. The findings underscore the importance of plaque accretion as a risk factor that is both modifiable and is of the greatest danger in an anatomically favorable population. To sum up, a multidisciplinary approach to cleft care can take place in the future with early PCR-based microbial screening, which can be used to provide timely and targeted preventive interventions with a decisive chance of reducing long-term periodontal morbidity and impoving oral health outcomes in children with cleft lip and palate. These findings highlight the clinical importance of early detection of periodontal pathogens in children with cleft lip and palate. The results emphasize plaque accumulation as a key modifiable risk factor and support the integration of preventive strategies and microbial screening into multidisciplinary cleft care to reduce long-term periodontal complications.

## Figures and Tables

**Table 1:** Oligonucleotide primer sequences employed for species-specific PCR detection of periodontal pathogens.

Target Microorganism	Target Gene	Primer Sequence (5′–3′)	Expected Amplicon Size (bp)
*Aggregatibacter actinomycetemcomitans*	16S rRNA	Forward: AAGGTCGACACGGTCCAGA Reverse: CAACTCTAGCACGAAAGCCA	557
*Porphyromonas gingivalis*	16S rRNA	Forward: AGGCAGCTTGCCATACTGCG Reverse: ACTGTTAGCAACTACCGATGT	404

**Table 2:** Demographic characteristics, cleft phenotype distribution, and periodontal status of the study population (n = 110).

Variable	n (%)
Age (years), mean ± SD	10.8 ± 2.6
Male sex	62 (56.4)
Female sex	48 (43.6)
Cleft lip (CL)	21 (19.1)
Cleft palate (CP)	34 (30.9)
Cleft lip and palate (CLP)	55 (50.0)
Moderate–high plaque index	75 (68.2)
Gingival inflammation present	80 (72.7)

**Table 3:** Molecular detection profile and co-colonization status.

Detection Status	N (%)
*A. actinomycetemcomitans only*	28 (25.5)
*P. gingivalis only*	14 (12.7)
Dual colonization	24 (21.8)
No pathogen detected	44 (40.0)

**Table 4:** Prevalence of periodontal pathogens according to demographic and cleft-related variables.

Variable	*A. actinomycetemcomitans* n (%)	*P. gingivalis* n (%)	*p*-Value
Age 6–8 years	12 (34.3)	5 (14.3)	0.004
Age 9–11 years	18 (48.6)	11 (29.7)	
Age 12–15 years	22 (61.1)	22 (61.1)	
Male	30 (48.4)	22 (35.5)	0.81
Female	22 (45.8)	16 (33.3)	
*CL*	7 (33.3)	4 (19.0)	0.018
*CP*	14 (41.2)	9 (26.5)	
*CLP*	31 (56.4)	25 (45.5)	

**Table 5:** Comparison of clinical periodontal parameters by pathogen detection status.

Parameter	Negative	Single-Positive	Dual-Positive	*p*-Value
Plaque Index	1.21 ± 0.34	1.78 ± 0.41	2.12 ± 0.39	<0.001
Gingival Index	1.08 ± 0.29	1.65 ± 0.37	2.01 ± 0.33	<0.001
Probing Depth (mm)	2.3 ± 0.4	3.1 ± 0.6	3.8 ± 0.7	<0.001

**Table 6:** Multivariable logistic regression analysis of predictors for periodontal pathogen detection.

Predictor	Adjusted OR	95% CI	*p*-Value
AGE ≥ 12 Years	3.41	1.62–7.18	0.001
Moderate–High Plaque	4.63	2.11–10.17	<0.001
CLP Phenotype	2.87	1.34–6.14	0.007
Male Sex	1.12	0.58–2.16	0.74

## Data Availability

The original contributions presented in this study are included in the article. Further inquiries can be directed to the corresponding author.
